# A novel nomogram model to predict in-hospital mortality in patients with acute type A aortic dissection after surgery

**DOI:** 10.1186/s13019-024-02921-6

**Published:** 2024-06-24

**Authors:** Yifei Zhou, Rui Fan, Hongwei Jiang, Renjie Liu, Fuhua Huang, Xin Chen

**Affiliations:** 1 School of Medicine, Southeast University, Nanjing, Jiangsu 210009 China; 2 The Department of Thoracic and Cardiovascular Surgery, Nanjing First Hospital, Nanjing Medical University, Changle Road 68, Nanjing, Jiangsu 210006 People’s Republic of China

**Keywords:** Acute type a aortic dissection, Mortality, Risk factor analysis, Nomogram

## Abstract

Acute type A aortic dissection is a dangerous disease that threatens public health. In recent years, with the progress of medical technology, the mortality rate of patients after surgery has been gradually reduced, leading that previous prediction models may not be suitable for nowadays. Therefore, the present study aims to find new independent risk factors for predicting in-hospital mortality and construct a nomogram prediction model. Methods: The clinical data of 341 consecutive patients in our center from 2019 to 2023 were collected, and they were divided into two groups according to the death during hospitalization. The independent risk factors were analyzed by univariate and multivariate logistic regression, and the nomogram was constructed and verified based on these factors. Results: age, preoperative lower limb ischemia, preoperative ***activated partial thromboplastin time (APTT)***, preoperative platelet count, ***Cardiopulmonary bypass (CPB)*** time and postoperative ***acute kidney injury (AKI)*** independently predicted in-hospital mortality of patients with acute type A aortic dissection after surgery. The ***area under the receiver operating characteristic curve (AUC)*** for the nomogram was 0.844. The calibration curve and decision curve analysis verified that the model had good quality. Conclusion: The new nomogram model has a good ability to predict the in-hospital mortality of patients with acute type A aortic dissection after surgery.

## Introduction

Aortic dissection is a highly lethal disease and is classified into type A and type B aortic dissection according to Stanford criteria [1]. Among them, acute type A aortic dissection (ATAAD) often leads to death due to its rapid progression, the mortality rate can reach 90% if not treated in time [2]. Surgical treatment is often the first choice, however, despite advances in surgical techniques, the postoperative mortality rate for type A aortic dissection can still be as high as 20% [3].

Given the high mortality rate of this kind of disease, it is critical to identify high-risk patients before surgery. Unfortunately, there are few studies on the prediction model of death during hospitalization for acute type A aortic dissection. Bleeding-related complications are associated with postoperative death in patients with acute type A aortic dissection, it has also been confirmed that acute type A aortic dissection is associated with coagulation dysfunction [4]. None of the previous studies included coagulation indicators in their models. Platelets play an important role in the blood clotting mechanism. In recent years, some platelet-related predictors such as ***SII (systemic immune-inflammation index)***, SCI (systemic coagulation-inflammation index), and ***PLR (Platelet to lymphocyte ratio)*** have shown favorable predictive value in clinical outcomes [5–7]. These indicators not only reflect the level of inflammation but also reflect the coagulation function to a certain extent. Therefore, whether the coagulation indicators can effectively predict postoperative death in patients with acute type A aortic dissection is particularly important. Based on this, we investigated the value of preoperative coagulation index such as platelet count and APTT in predicting postoperative death in patients with acute type A aortic dissection and constructed a nomogram model to predict in-hospital death in patients with acute type A aortic dissection.

## Methods

### Study population

This study was approved by the Ethics Committee of Nanjing First Hospital (approval number: KY20220425-05). We selected patients with acute type A aortic dissection who underwent emergency surgical procedures at our center between January 2019 and December 2023. ***All patients underwent Sun’s procedure.*** The inclusion criteria were as follows: (1) age > 18 years; (2) diagnosis of type A aortic dissection was confirmed by computed tomographic angiography (CTA); (3) emergency surgical treatment performed within 24 h of onset. The exclusion criteria were as follows: (1) chronic aortic dissection or time interval from symptom onset to surgery exceeding 24 h; (2) a history of cardiovascular surgery; (3) iatrogenic aortic dissection that occurs during other types of cardiovascular surgery; (4) patients who had chronic hepatic and renal insufficiency or autoimmune diseases; (5) traumatic aortic dissection and (6) incomplete case data.

### Definitions

Preoperative lower limb ischemia is defined as a diminished pulse or pulselessness with pain, pallor, paresthesia, poikilothermia, or paralysis at the involved extremity [8], postoperative prolonged mechanical ventilation is defined as the duration of mechanical ventilation exceeding 24 h after surgery, postoperative AKI is defined as an elevation of serum creatinine level 3-fold greater than baseline or a new requirement for hemodialysis, postoperative gastrointestinal bleeding is defined as specific bleeding points under endoscope.

### Data collection

Clinical data of enrolled patients were retrieved from the electronic medical record system, including baseline characteristics (age, gender, etc.), ***preoperative chronic diseases history (hypertension, diabetes, coronary artery disease, etc.)***, preoperative blood examines; intraoperative data (cardiopulmonary bypass time, aortic cross-clamp time, etc.), and the postoperative clinical outcomes include in-hospital death and postoperative complications. ***We used multiple imputation techniques to account for missing values in the collected data.***

### Statistical analysis

All statistics were analyzed by SPSS version 26 (SPSS Inc., Chicago, IL, USA) and R version 4.1.2 (R Foundation for Statistical Computing, Vienna, Austria). The normality of the data distribution was tested using the Kolmogorov–Smirnov test. Continuous variables were presented as mean ± standard deviation or median (interquartile range) depending on whether it is according to a normal distribution. Unpaired Student’s t-tests were conducted for normally distributed continuous variables, while the Mann-Whitney U test was used for non-normally distributed continuous variables. Categorical variables were presented as percentages and analyzed with the chi-square tests. A *p* < 0.05 was considered statistically significant. All enrolled patients were divided into two groups based on whether in-hospital death occurred, variables that showed significant differences between the two groups were subjected to univariate logistic regression analysis. Multivariate logistic regression analysis was conducted to select independent predictors and prediction models, then a predictive nomogram was constructed based on statistical significance. The prediction accuracy of the nomogram was tested using the concordance index (C-index) and areas under the curve (AUC). The calibration curve, and internal validation by bootstrap repetitions 1000 times were used to evaluate the prediction model. Additionally, a decision curve analysis (DCA) curve was employed to determine the threshold of net benefit for the prediction. ***We used the Hosmer and Lemeshow test to check the goodness of fit of the data. In addition, sensitivity analysis was used to ensure the accuracy and stability of the model.***

## Results

### Baseline characteristics

**Fig. 1:**
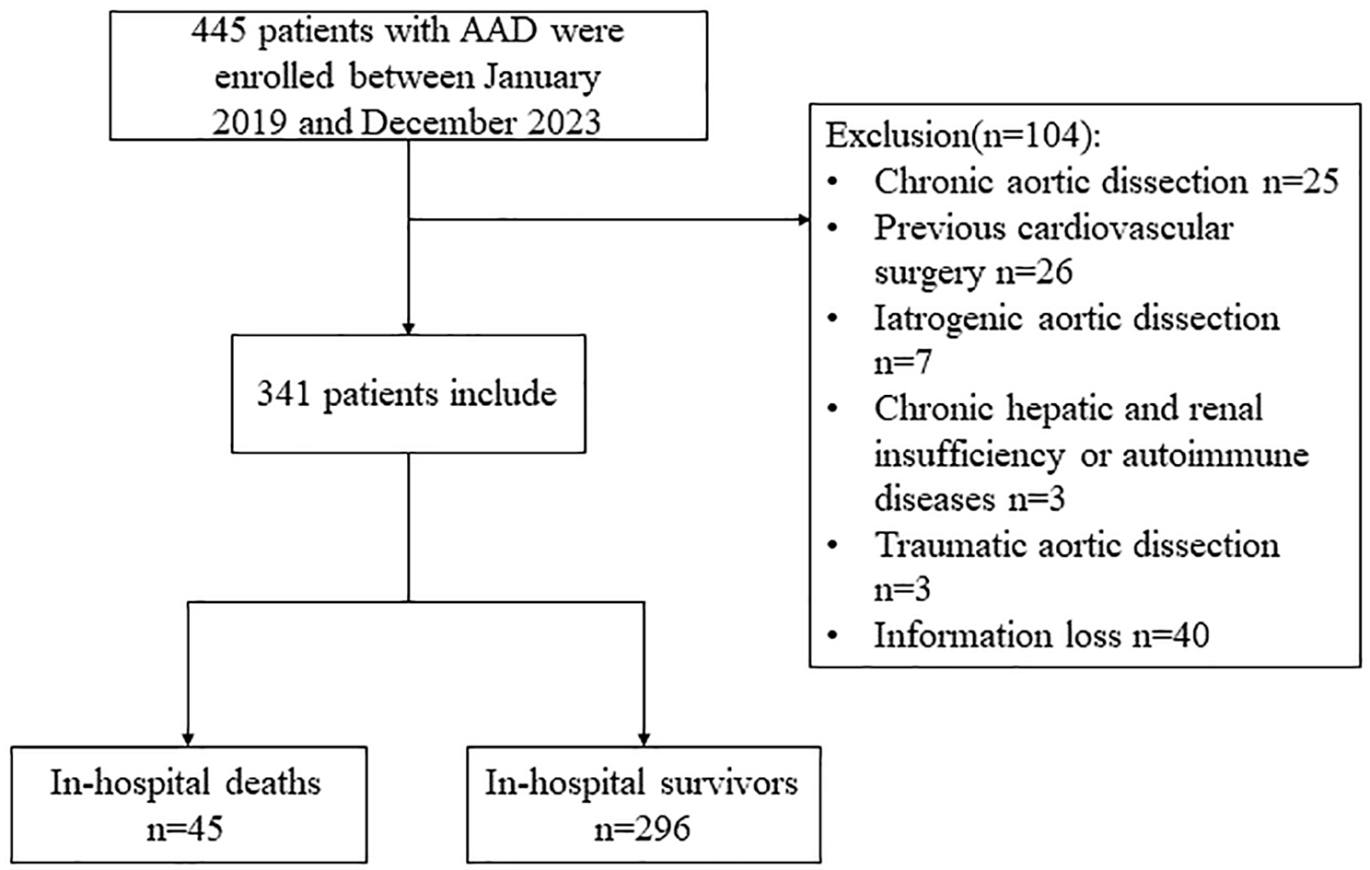
Flow chart of the research

Among the enrolled 445 patients, 104 patients were excluded as shown in the flow chart (Fig. 1). The remaining 341 patients were included for further analysis, 45 (13.2%) occurred in-hospital deaths. Notably, significant differences were observed in terms of age, preoperative shock, preoperative lower limb ischemia, preoperative platelet count, APTT, CPB time, concomitant ***coronary artery bypass grafting (CABG)*** procedures, prolonged postoperative mechanical ventilation, postoperative AKI, postoperative reintubation and postoperative gastrointestinal bleeding (*P* < 0.05), while no statistically significant disparities were found among other parameters (Table 1).

**Table 1:** Demographics and laboratory examinations of all patients with acute type A aortic dissection

Variables	Survival(*n* = 296)	Death(*n* = 45)	*P* value
Age (years)	54.2 ± 12.5	60.2 ± 12.3	0.003*
BMI (kg/m2)	26.4 ± 4.0	26.5 ± 4.5	0.938
Male, n (%)	225(76.0)	29(64.4)	0.098
Drinking history, n (%)	97(32.8)	11(24.4)	0.263
Smoking history, n (%)	154(52.0)	21(46.7)	0.503
Hypertension, n (%)	243(82.0)	37(82.2)	0.983
Diabetes mellitus, n (%)	31(10.5)	3(6.7)	0.427
COPD, n (%)	6(2.0)	2(4.4)	0.318
Cerebral infarction, n (%)	14(4.7)	4(8.9)	0.245
CHD, n (%)	25(8.4)	6(13.3)	0.288
Preoperative shock, n (%)	5(1.7)	10(22.2)	< 0.001*
Preoperative cardiopulmonary resuscitation, n (%)	3(1.0)	2(4.4)	0.074
AF, n (%)	6(2.0)	0	0.335
Preoperative lower limb ischemia, n (%)	57(19.3)	19(42.2)	0.001*
Platelet count (10^9^/L)	175.5 ± 58.2	154.1 ± 48.3	0.020*
PT (s)	12.1 ± 1.3	12.5 ± 1.6	0.207
White blood cells (10^9^/L)	13.1 ± 4.2	13.7 ± 4.4	0.342
Hemoglobin (g/L)	132.4 ± 18.8	131.1 ± 20.2	0.651
Creatine kinase (U/L)	97(43.5-150.5)	92(48.5-135.5)	0.434
Urea nitrogen (mmol/L)	6.9 ± 2.9	7.0 ± 1.9	0.868
Creatinine (µmol/L)	95.7 ± 93.4	89.7 ± 41.4	0.675
APTT (s)	28.7 ± 4.7	30.2 ± 6.0	0.045*
Fibrinogen (g/L)	2.7 ± 1.5	2.6 ± 1.5	0.630
Concomitant CABG procedures, n (%)	24(8.1)	10(22.2)	0.003*
CPB time (min)	174.1 ± 39.1	198.2 ± 65.8	0.020*
Aortic cross-clamp time (min)	98.1 ± 27.6	101.4 ± 30.0	0.463
Postoperative prolonged mechanical ventilation, n (%)	125(42.2)	32(71.1)	< 0.001*
Postoperative AKI, n (%)	49(16.6)	23(51.1)	< 0.001*
Postoperative reintubated, n (%)	20(6.8)	11(24.4)	< 0.001*
Postoperative paraplegia, n (%)	9(3.0)	3(6.7)	0.219
Postoperative gastrointestinal bleeding, n (%)	10(3.4)	8(17.8)	< 0.001*

BMI: Body Mass Index; COPD: Chronic Obstructive Pulmonary Disease; CHD: Coronary Heart Disease; AF: Atrial Fibrillation; PT: Prothrombin Time; APTT: Activated Partial Thromboplastin Time; CABG: Coronary Artery Bypass Grafting; CPB: Cardiopulmonary Bypass; AKI: Acute Kidney Injury. *P < 0.05

### Independent predictors of in-hospital death

Univariate and multivariate logistic analyses were applied to obtain potential predictors for in-hospital deaths. Finally, age (OR: 1.05; 95%CI:1.016 to 1.085; *P* = 0.003), preoperative lower limb ischemia (OR: 2.76; 95%CI: 1.197 to 6.353; *P* = 0.017), APTT (OR: 1.07; 95%CI: 1.001 to 1.145; *P* = 0.046), platelet count (OR: 0.99; 95%CI: 0.983 to 0.999; *P* = 0.02), CPB time (OR: 1.01; 95%CI: 1.001 to 1.019; *P* = 0.029), and postoperative AKI (OR: 4.62; 95%CI: 1.994 to 10.706; *P* < 0.001) were identified as independent predictors for in-hospital deaths in patients with acute type A aortic dissection (Table 2).

**Table 2:** Univariable and multivariable logistic regression analysis of risk factors for in-hospital mortality of patients with acute type A aortic dissection after surgery

Variables	Univariate			Multivariate		
	OR	95%CI	*P*	OR	95%CI	*P*
Age	1.041	1.013–1.070	0.003	1.05	1.016–1.085	0.003
Preoperative shock	3.575	1.163–10.994	0.026			
Preoperative lower limb ischemia	3.064	1.586–5.919	0.001	2.757	1.197–6.353	0.017
APTT	1.058	1-1.118	0.048	1.071	1.001–1.145	0.046
Platelet count	0.993	0.986–0.999	0.021	0.991	0.983–0.999	0.02
Concomitant CABG procedures	3.238	1.430–7.333	0.005			
CPB time	1.011	1.004–1.017	0.001	1.01	1.001–1.019	0.029
Postoperative prolonged mechanical ventilation	3.367	1.698–6.678	0.001			
Postoperative AKI	5.27	2.724–10.196	< 0.001	4.62	1.994–10.706	< 0.001
Postoperative reintubated	4.465	1.971–10.111	< 0.001			
Postoperative gastrointestinal bleeding	6.184	2.296–16.654	< 0.001			

APTT: Activated Partial Thromboplastin Time; CABG: Coronary Artery Bypass Grafting; CPB: Cardiopulmonary Bypass; AKI: Acute Kidney Injury

### ROC analysis of predictors

We carried out ***receiver operating characteristic curve (ROC)*** analysis on the variables selected by logistic regression respectively. As shown in Table 3, their AUCs and 95% CIs were Age: 0.638 (95% CI: 0.548–0.727), PLT count: 0.611(95% CI: 0.531–0.691), APTT: 0.593(95% CI: 0.510–0.676), Postoperative AKI: 0.673(95% CI: 0.580–0.765), CPB time: 0.595(95% CI: 0.490–0.699), Preoperative lower limb ischemia: 0.615(95% CI: 0.521–0.709). We also combine all the predictors and obtain a better AUC (0.844, 95% CI: 0.784–0.905).

**Table 3:** ROC analysis for each predictor

Variables	AUC	95%CI	*P* value
Age	0.638	0.548–0.727	0.003
Platelet count	0.611	0.531–0.691	0.016
APTT	0.593	0.510–0.676	0.044
Postoperative AKI	0.673	0.580–0.765	< 0.001
CPB time	0.595	0.490–0.699	0.045
Preoperative lower limb ischemia	0.615	0.521–0.709	0.013
Combine	0.844	0.784–0.905	< 0.001

APTT: Activated Partial Thromboplastin Time; AKI: Acute Kidney Injury; CPB: Cardiopulmonary Bypass

### Development of the nomogram model

**Fig. 2:**
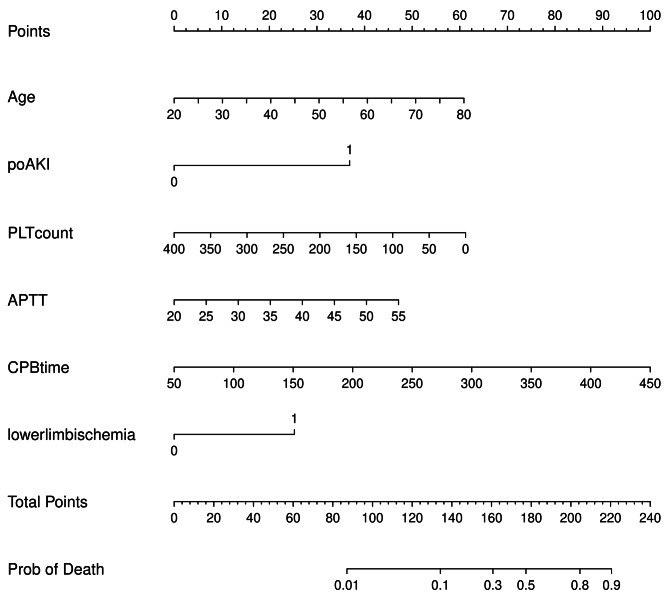
Nomogram model for predicting the risk of in-hospital death in patients with acute type A aortic dissection. ***All these variables were assigned a score on the points scale, and a total point was obtained and located on the Total points scale by accumulating points for 6 variables***, which could be used to estimate the probability of the risk. poAKI, postoperative acute kidney injury; PLT, platelet; APTT, activated partial thromboplastin time; CPB, cardiopulmonary bypass

We use the six independent in-hospital mortality predictors mentioned above to construct the predictive nomogram (Fig. 2). Each variable has a corresponding score, and the total score is obtained by adding the scores for all variables. The higher the total score, the greater the patient’s risk of in-hospital deaths.

### The performance of the nomogram

**Fig. 3:**
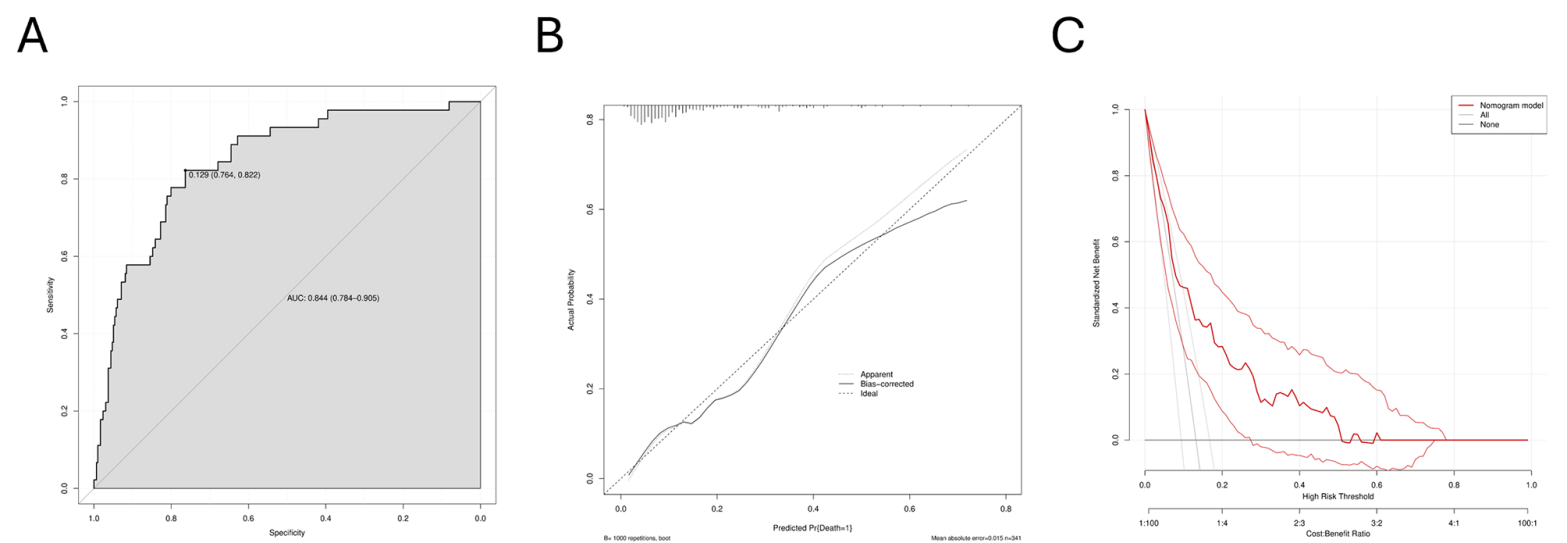
Evaluation of the prediction model. (**A**) ROC curve for evaluating the model’s discrimination performance. The AUC of the ROC curve is 0.844 (95% CI, 0.784–0.905). ROC, receiver operating characteristic; AUC, area under the curve. (**B**) The calibration curve displayed the deviation between predicted and observed probability. Repetitions 1000 times were performed for internal validation. (**C**) DCA for the predictive nomogram, the gray line indicates the assumption that all patients with a diagnosis of acute type A aortic dissection, and the black line represents the assumption that no patient with a diagnosis of acute type A aortic dissection. The DCA indicated that the predictive nomogram had higher overall net benefits in predicting acute type A aortic dissection. DCA, decision curve analysis; CI, confidence interval

**Fig. 4:**
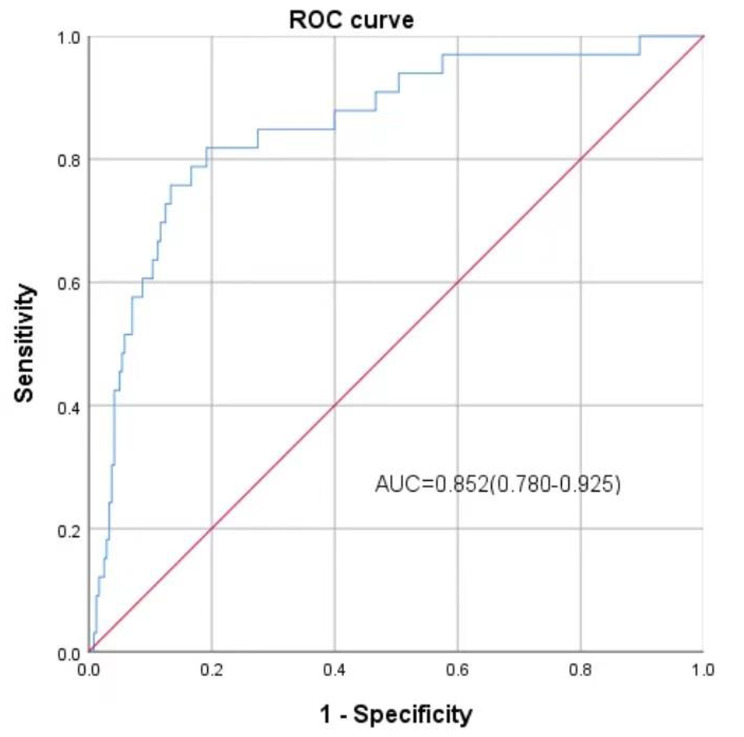
ROC curve evaluation in the nomogram model completely excluding cases with missing values

C-index, also expressed as AUC was used to assess the discriminatory capacity of the model, the predictive nomogram validated a discriminative capacity of AUC: 0.844 (95% CI: 0.784–0.905) (Fig. 3A). In addition, the calibration curve with an internal validation of 1000 times repetitions was shown (Fig. 3B), indicating good consistency between predicted and observed probability. The decision curve analysis (Fig. 3C) indicated that compared with the “no intervention” or “intervention for all” strategy, using the nomogram model could get more clinical net benefits. *The Hosmer-Lemeshow test obtained a* *p*-value of 0.554, indicating that the current model is statistically acceptable despite not being an ideal fit. Sensitivity analyses were performed to evaluate the accuracy and stability of the model. After completely excluding cases with missing values, the area under the ROC curve was 0.852 (95%CI: 0.780–0.925) (Fig. 4*). The results were consistent with the conclusions obtained by the multiple imputation method in this study, and both of them had good predictive power.*

## Discussion

In this study, a prediction model was constructed using several clinically accessible indicators to evaluate the risk of in-hospital deaths. In recent years, with advances in surgical techniques, in-hospital mortality in patients with acute type A aortic dissection has been greatly reduced. In the present study, the in-hospital mortality was 13.19%. A survey between 2014 and 2018 showed a 33.65% in-hospital mortality [9]. In addition, another study indicated in-hospital mortality was around 18% [10]. Such changes suggest that previous predictive models are not suitable for nowadays. For this, new models need to be built. Through univariate and multivariate logistic regression analysis, we identified age, preoperative lower limb ischemia, preoperative platelet count, preoperative APTT, CPB time, and postoperative AKI are independent risk factors for in-hospital mortality in patients with acute type A aortic dissection. A novel prediction model and nomogram were established by these predictors and the model performed well in discrimination, calibration, and clinical utility.

Our study confirms that patients with acute type A aortic dissection with preoperative lower limb ischemia have higher in-hospital mortality, this phenomenon is consistent with previous studies [11, 12]. Lower limb ischemia often leads to serious postoperative complications; thus, it can identify those at high risk of in-hospital adverse events [13], this may be due to the release of toxins from ischemic necrosis, which affects liver and kidney function, and then leads to systemic organ failure, leading to adverse outcomes eventually. Extra-anatomic bypass grafting during aortic repair can effectively solve the problem [14]. The study by Natour et al. noted asymptomatic graft occlusion in all follow-up patients who underwent extra-anatomic bypass [15], which may be attributed to competing blood flow from a remodeling autologous arterial system. However, it is interesting that this does not affect the long-term survival rate of patients, which may suggest that rapid restoration of perfusion in lower limb ischemia can achieve good results. ***In addition to lower limb ischemia, coronary ischemia and prolonged postoperative mechanical ventilation time caused by cerebral ischemia are other manifestations of malperfusion. However, multivariate logistic regression showed that coronary ischemia and cerebral ischemia were not independent risk factors for postoperative death in patients with acute type a aortic dissection. This may be due to the collateral circulation of the coronary artery and the cerebral protective effect of intraoperative hypothermia. Further research is needed to confirm this hypothesis.***

Previous studies of type A aortic dissection have focused on vascular inflammation [16, 17], these indicators of systemic inflammation also include indicators related to blood coagulation, such as platelet count, in addition, coagulopathy has also been demonstrated to be associated with type A aortic dissection [4]. The coagulation pathway is usually composed of endogenous and exogenous pathways. After the occurrence of aortic dissection, the exogenous coagulation function is activated, leading to a large consumption of coagulation substances, including platelets. The inflammatory response caused by thrombus formation in the false lumen of aortic dissection can promote platelet activation, further accelerate the consumption of platelets, and ultimately affect the prognosis of patients [18]. Research has also suggested that platelet function and activity were closely related to cervicocephalic artery dissections [19]. These conclusions are consistent with our findings that low preoperative platelet count is an independent risk factor for in-hospital mortality in patients with acute type A aortic dissection after surgery.

Activated partial thromboplastin time (APTT) is also involved in the coagulation pathway, it is often used to reflect the activity of coagulation factors in the endogenous coagulation pathway. One study demonstrated that higher values of APTT were found in the deceased group of patients with type A aortic dissection [20]. Therefore, we also collected the preoperative blood APTT levels of the patients and finally concluded that the patients who died during hospitalization had higher APTT levels.

CPB time was independently correlated to in-hospital mortality in this study, and this association was consistent with some previous study results in patients undergoing cardiac surgery [21, 22]. Preventza et al. suggested that in patients with reoperations on the total aortic arch, a longer CPB time is more likely to result in composite adverse outcomes [23]. It can also predict poor outcomes after on-pump coronary artery bypass surgery [24]. Besides, CPB can cause systemic inflammation and multiple disorders of the clotting and fibrinolytic systems [25], as the research showed that longer CPB time may result in massive bleeding in ATAAD patients who underwent emergent aortic repair [26], and abnormal coagulation function associated with bleeding affects in-hospital mortality as mentioned above [4]. Platelet defects after cardiopulmonary bypass further aggravate coagulation dysfunction and eventually lead to adverse events [27].

AKI after acute type A aortic dissection surgery is a relatively common complication and has been proven to affect both short- and long-term outcomes. The incidence of postoperative AKI in our study was 21%, which is consistent with previous studies [28]. In addition, the severity of AKI is positively correlated with mortality, and in one study, stage 3 AKI after surgery was associated with a decreased postoperative survival rate [29].

There are also some limitations in this study. First, this is a single-center retrospective study, which needs further prospective studies to prove. Secondly, due to the small sample size included in this study, only the prediction model was internally validated. In the next study, multi-center data could be included for external validation. ***In addition, the consumption of coagulation factors and platelets may be related to the range of the patient’s dissection, so further studies can be conducted.***

## Conclusion

Our study proposed a new nomogram model for predicting in-hospital mortality in patients with acute type A aortic dissection after surgery and verified that the nomogram model has good predictive ability, which can be used as a new reference for clinicians.

## Data Availability

No datasets were generated or analysed during the current study.

## Abbreviations

AKI: acute kidney injury
AUC: Area under the receiver operating characteristic curve
CI: Confidence interval
CPB: Cardiopulmonary bypass
PLT: platelet
APTT: activated partial thromboplastin time
OR: Odds ratio
